# Tandem RNA isolation reveals functional rearrangement of RNA-binding proteins on *CDKN1B/p27*^Kip1^ 3’UTRs in cisplatin treated cells

**DOI:** 10.1080/15476286.2019.1662268

**Published:** 2019-09-16

**Authors:** Valentina Iadevaia, Maikel D. Wouters, Alexander Kanitz, Ana M. Matia-González, Emma E. Laing, André P. Gerber

**Affiliations:** a Department of Microbial Sciences, School of Biosciences and Medicine, Faculty of Health and Medical Sciences, University of Surrey, Guildford, GU2 7XH, UK; b Biozentrum, University of Basel, Basel, Switzerland

**Keywords:** Post-transcriptional gene regulation, RNA-binding protein, mRNA stability, drug response, p27, KHSRP

## Abstract

Post-transcriptional control of gene expression is mediated via RNA-binding proteins (RBPs) that interact with mRNAs in a combinatorial fashion. While recent global RNA interactome capture experiments expanded the repertoire of cellular RBPs quiet dramatically, little is known about the assembly of RBPs on particular mRNAs; and how these associations change and control the fate of the mRNA in drug-treatment conditions. Here we introduce a novel biochemical approach, termed tobramycin-based tandem RNA isolation procedure (tobTRIP), to quantify proteins associated with the 3ʹUTRs of cyclin-dependent kinase inhibitor 1B (*CDKN1B/p27^Kip1^*) mRNAs *in vivo*. P27^Kip1^ plays an important role in mediating a cell’s response to cisplatin (CP), a widely used chemotherapeutic cancer drug that induces DNA damage and cell cycle arrest. We found that *p27^Kip1^* mRNA is stabilized upon CP treatment of HEK293 cells through elements in its 3ʹUTR. Applying tobTRIP, we further compared the associated proteins in CP and non-treated cells, and identified more than 50 interacting RBPs, many functionally related and evoking a coordinated response. Knock-downs of several of the identified RBPs in HEK293 cells confirmed their involvement in CP-induced *p27* mRNA regulation; while knock-down of the KH-type splicing regulatory protein (KHSRP) further enhanced the sensitivity of MCF7 adenocarcinoma cancer cells to CP treatment. Our results highlight the benefit of specific *in vivo* mRNA-protein interactome capture to reveal post-transcriptional regulatory networks implicated in cellular drug response and adaptation.

## Introduction

Post-transcriptional regulation of gene expression plays a pivotal role in maintaining cellular homoeostasis. As aberrant control of cellular homoeostasis contributes to cancer, this may implicate post-transcriptional processes in cancer progression or treatment [1,2]. Post-transcriptional control is mainly exerted by the interaction of a specific target mRNA with one or more RNA-binding proteins (RBPs) or non-coding RNAs (ncRNAs) such as microRNAs (miRNAs), which occur preferentially in the untranslated regions (UTRs) of cytoplasmic mRNA and in a combinatorial fashion [3,4]. Importantly, the dynamic assembly of RBPs and/or ncRNAs orchestrates all aspects of an mRNA’s life; from RNA processing events in the nucleus (i.e., splicing), to the control of mRNA stability, translation or localization to the cytoplasm. Recently, global experimental exploration of RBPs in diverse organisms has become popular, with some studies exploring the dynamics of mRNA-RBP association upon changing environmental conditions [5–8]. However, changes in specific mRNA–RBP interactions between conditions are currently understudied. This is likely because the applicability of existing experimental approaches is limited. Current approaches involve either the affinity purification of mRNAs with specifically designed antisense oligonucleotides (ASOs) (e.g., [9–11]) or the recovery of tagged mRNAs via specific ligands or proteins [12,13]. The level of specificity and efficiency of target mRNA capture is a common limitation of these approaches, especially for *in vivo* applications, as well as low mRNA copy number. We thus developed a novel two-step tobramycin aptamer-based purification strategy, termed tobTRIP, to identify proteins bound to the 3ʹ untranslated regions (UTR) of specific mRNAs. The approach complements our previous tandem RNA affinity isolation procedure (TRIP) by using ASOs to capture endogenous mRNAs [10,11].

Cisplatin (CP; *cis*-diamminedichloroplatinum(II)) is a widely used chemotherapeutic agent for treating human cancers [14–16]. CP binds to DNA and forms intra- and inter-strand DNA cross-links as well as mono adducts, which mediate cytotoxic effects by interfering with transcription and replication and ultimately lead to the induction of cell death through apoptosis [14–16]. Importantly, several studies monitoring the effects of CP on gene expression suggest the involvement of post-transcriptional regulatory mechanisms, although the specific mediators remain unknown [17–19]. Cell-cycle control is one of the major checkpoints for DNA repair and a key process for tumour progression and maintenance [20,21]. Transition from G1 to S phase of the cell cycle is controlled by p27 (also referred to CDKN1B or Kip1), a negative regulator of CDK2 and cyclin E expression that prevents the entry of cells into S phase [22]. p27 has been long thought to mainly act as a tumour suppressor since it can halt the cell-cycle and promotes apoptosis [23]. Conversely, p27 has also been shown to have anti-apoptotic effects by protecting cells from cytotoxic stress [24]. Most relevant to this work is that there is evidence for post-transcriptional control of p27 with potential implications in cancer therapy. For example, p27 is induced in non-small cell lung cancer (NSCLC) tumours and shown to be post-transcriptionally controlled by the RBP hnRNPA0, which promotes DNA repair and makes cells tolerant to chemotherapy [24]. As such, p27 can drive tumorigenesis [25], with high levels of p27 correlated with CP resistance (e.g., [26]). Nevertheless, the reported implications of p27 for tumour growth and drug resistance are diverse, possibly reflecting the complexity and differences in the cellular composition of different tumour types.

To further our understanding of RBP-mediated post-transcriptional regulation of *p27* mRNAs in response to CP drug response, we used tobTRIP to investigate the binding of RBPs before and after CP treatment of HEK293 cells, which have been shown to be sensitive to CP [27]. We established that *p27* mRNA is stabilized upon CP treatment, while translation is generally repressed. Applying tobTRIP, we could identify a network of RBPs associated with the 3ʹUTR of *p27*. Knock-down of selected RBPs inhibited induction of *p27* mRNA levels upon CP treatment in HEK293 cells, while KHSRP knock-down enhanced the sensitivity of MCF7 adenocarcinoma cancer cells to CP treatment. Our results therefore highlight the importance of post-transcriptional regulation in drug response and KHSRP for modulation of drug sensitivity.

## Results

### *Post-transcriptional regulation of* p27 *in CP-treated HEK293 cells*

**Figure 1.**
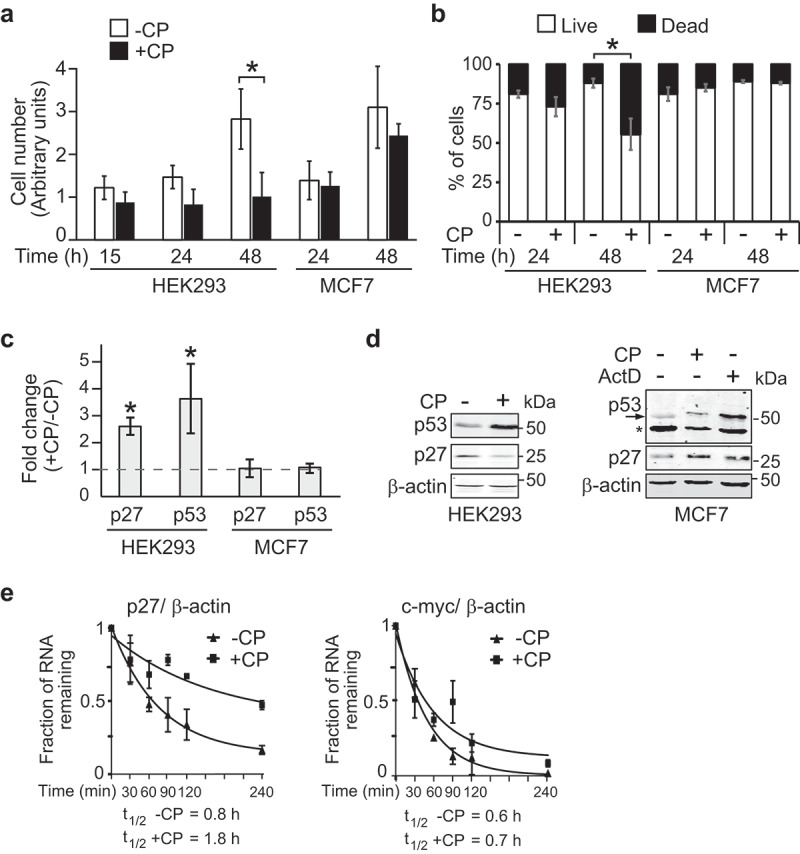
CP affects cell proliferation and *p27* mRNA stability in HEK293 but not in MCF7 cells. (a) Proliferation assay of cells treated (+CP; 20 µM) or not treated (-CP) with CP for the indicated time periods. (b) Trypan blue cell viability assay. Cells were counted (n = 3) and the result reported as a percentage of dead versus live cells in a bar plot. (c) Relative changes of *p27* and *p53* mRNAs in CP-treated (20 µM of CP for 15 h) compared to untreated (-CP) HEK293 and MCF7 cells as measured by RT-qPCR normalized to *β-actin* mRNA. Error bars represent the standard error of the mean (SEM), n = 3. *P < 0.05. (d) Immunoblot analysis with antibodies against the specified proteins. (e) HEK293 cells were treated with 20 µM CP for 15 h prior to the addition of 2 µg/ml of ActD for 30, 60, 90, 120, and 240 min. The half-life of *p27* and *c-myc* mRNAs relative to *β-actin* was determined by RT-qPCR considering ‘one phase decay equation’ implemented in GraphPad Prism. Error bars represent SEM, n = 3.

To evaluate the inhibition of cell-growth by CP we compared cell proliferation of HEK293 and MCF7 cells in the presence (20 µM) and absence of CP. As reported [27], we observed a significant reduction in cell proliferation of CP-treated HEK293 after 24 h (h) (Figure 1(a)). Contrarily, MCF7 cells, a human breast adenocarcinoma cell line considered to be relatively resistant to CP treatment [28], were still proliferating after 24 h in the presence of CP with some slight growth reduction after 48 h. Furthermore, the fraction of dead cells was significantly higher in CP-treated HEK293 cells, whereas no significant differences were observed for MCF7 cells (Figure 1(b)). These results are in line with increased cytotoxic effects of CP in HEK293 compared to MCF7 cells.

Since expression of p27 has been linked to the DNA damage response, which can be triggered by CP, we compared p27 mRNA and protein levels in CP-treated *versus* untreated cells (Figure 1(c,d); uncropped immunoblots are provided in the Supplemental Material). Thus, we focused on 15 h CP treatment to minimize potential secondary effects induced by defects in cellular proliferation that can occur after prolonged exposure to CP. *p27* mRNA levels were increased after 15 h of CP treatment, whereas protein levels were slightly reduced in HEK293 cells; the latter in agreement with previous data obtained in other cells (i.e., MHM, U2OS and S4 cells) [29]. Contrarily, no significant changes in p27 mRNA and protein levels were observed in MCF7 cells. CP treatment leads to the activation of members of the tumour protein p53 family of transcription factors, which, in turn, alter the expression of downstream target genes leading to increased DNA repair, cell-cycle arrest and eventually apoptosis [15,30]. Furthermore, it has been reported that p53 upregulates p27 expression in breast cancer [31]. As could be expected, p53 mRNA and protein levels were significantly elevated in CP-treated HEK293 cells after 15 h as compared to untreated cells (Figure 1(c,d)). Conversely, no substantial increase of p53 protein and mRNA levels was observed in MCF7 cells upon CP treatment for 15 h (Figure 1(c,d)), although extended application of CP for 48 h led to some increase of p53 levels (data not shown). Notably, p53 protein levels were increased in MCF7 cells exposed to 2 µg/ml of ActD for 3 h, a condition known to activate p53 [32] and confirms the integrity of the p53 pathway in MCF7 cells (Figure 1(d)). The observed different responses on p27 and p53 levels in HEK293 as compared to MCF7 upon CP treatment are therefore in line with the different sensitivities of cells to CP treatment and the associated physiological response, such as DNA damage response.

We next tested whether CP administration compromises translation, which may explain the observed slight reduction in p27 protein levels despite increased mRNA levels. An analysis of polysomal profiles obtained from CP-treated and untreated HEK293 cells showed an accumulation of 80S monosomes concomitant with a reduction of polysomes, indicative of general inhibition of protein synthesis (Supplemental Figure S1(a)). Moreover, *p27* mRNAs shifted from heavy polysomes to light polysomes upon CP treatment (Supplemental Figure S1(b)). However, similar shifts from heavy/lighter polysomes to subpolysomes were seen for tubulin (*TUBB*) and – even more pronounced – for *RPS18*, a terminal oligopyrimidine track (TOP) sequence-bearing mRNA known to be particularly affected in translation upon stress conditions [33]. We thus concluded that translation is generally inhibited upon CP treatment of cells and therefore not specific to *p27* mRNA.

We next wondered whether the application of CP induces the formation of stress-granules (SGs), which could explain the general inhibition of protein synthesis [34]. However, CP treatment did not lead to the visible formation of SGs after 15 h, indicating that CP-mediated inhibition of protein synthesis does not coincide with the formation of SGs (Supplemental Figure S2).

The increased *p27* mRNA levels in CP-treated HEK293 cells could be explained by reduced mRNA turnover and/or increased production through transcription. We therefore investigated whether the stability of *p27* mRNA is affected in CP-treated compared to untreated cells by addition of ActD as a potent inhibitor of transcription [32]. Indeed, we found that *p27* mRNA became more stable in CP-treated cells (*P* = 0.02, two-tailed student’s t-test), whereas no significant alterations in the stability were observed for *c-myc* mRNA (*P* = 0.23), a relatively unstable mRNA species [35] (Figure 1(e)). These results suggest that *p27* mRNA is specifically stabilized upon short (15 h) CP treatment, while its translation is slightly inhibited, likely through a global decrease in translation efficiency. However, it is important to note that increased mRNA stability does not exclude the possibility for increased transcription of *p27*, which could also contribute to the observed increase of *p27* mRNA levels in CP-treated cells. Moreover, whether the apparent paradox implemented by the observed increase of *p27* mRNA stability along its translation repression could relate to alternative functions of *p27* mRNAs, such as regulatory or epigenetic functions would need further investigation [36].

### P27 *gene expression is post-transcriptionally regulated by the 3ʹUTR of its mRNA*

**Figure 2.**
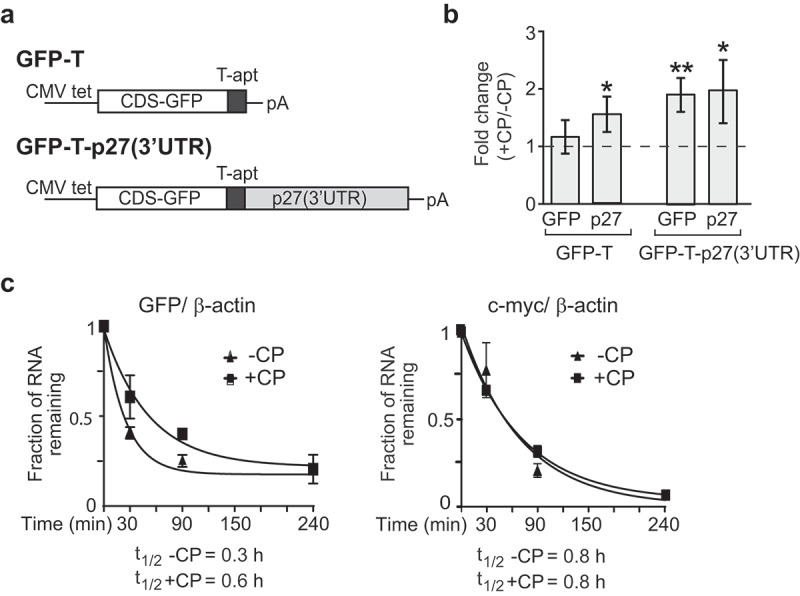
P27(3ʹUTR) reporter mRNA recapitulates post-transcriptional regulation in CP-treated cells. (a) Schematic representation of GFP-T and GFP-T-p27(3ʹUTR) reporter constructs. GFP-T cells lack exogenous *p27* 3ʹUTR sequences. (b) Changes in *GFP* and endogenous *p27* mRNAs in CP-treated (+CP) compared to untreated (-CP) GFP-T and GFP-T-p27(3ʹUTR) inducible cell lines. Transcript levels were quantified with RT-qPCR and normalized to *β-actin* mRNA. Error bars represent SEM, n = 3. *P < 0.05, **P < 0.01. (c) mRNA decay of GFP-T-p27(3ʹUTR) in untreated (-CP) and CP-treated (20 µM CP for 15 h) cells. The half-life of *GFP* and *c-myc* mRNAs was determined relative to *β-actin* with RT-qPCR and plotted as the mean, considering ‘one phase decay equation’ implemented in GraphPad Prism. Error bars represent SEM, n = 2.

Since mRNA stability is often controlled through RNA–protein interactions in the 3ʹUTR, we wanted to test whether the 3ʹUTR contributes to the stabilization of *p27* mRNAs in CP-treated HEK293 cells. Therefore, we generated HEK293 cell lines that allow tetracycline (tet)-inducible expression of the *p27* 3ʹUTR from a stable integrated plasmid. The integrated construct contains the coding sequence of green fluorescent protein (GFP), as well as the sequence for a novel RNA affinity tag, termed HAMMER2 (T-apt), located between the GFP coding sequence and the *p27* 3ʹUTR (Figure 2(a)). The HAMMER2 tag comprises a J6f1 aptamer, which efficiently interacts with tobramycin (Kd = 5 nM [37]), and is flanked by linker sequences in order to stabilize the local RNA aptamer structure, as well as restriction sites for DNA sub-cloning of alternate aptamers and 3ʹUTR sequences (construction and features of HAMMER2 and corresponding plasmids for generation of cell lines are outlined in the Supplemental Figure S3). The tet-inducible expression of GFP in stable cell lines containing (GFP-T-p27(3ʹUTR)) or lacking the 3ʹUTR of *p27* (GFP-T) was validated by fluorescence microscopy and immunoblot analysis (Supplemental Figure S4). The latter revealed that inclusion of the *p27* 3ʹUTR leads to reduction in GFP expression, indicating inhibitory functions of the 3ʹUTR for gene expression.

To test whether the observed increase in *p27* mRNA upon CP treatment can be recapitulated by its 3ʹUTR alone we treated GFP-T-p27(3ʹUTR) and GFP-T cells for 15 h with CP. CP treatment significantly increased the abundance of the GFP-T-p27(3ʹUTR) mRNA to levels similar to that of endogenously expressed *p27*, but not those of GFP-T mRNA lacking the *p27* 3ʹ-UTR (Figure 2(b)). Conversely, GFP protein levels remained constant or were slightly decreased in both GFP-T and GFP-T-p27(3ʹUTR) cells, in agreement with the previously observed general reduction of translation upon CP treatment (Supplemental Figure S4(b)). Finally, we measured the relative mRNA stability of the reporter GFP-T-p27(3ʹUTR) upon CP treatment. As previously seen with the endogenous *p27* mRNAs, we found substantial stabilization of the reporter mRNAs upon CP treatment (*P* = 0.037), while *c-myc* was not changed (*P* = 0.97)(Figure 2(c)). These results strongly suggest that (i) sequences in the 3ʹUTR of *p27* mediate, at least in part, the stabilization of the mRNA upon CP treatment, and that (ii) the reporter is valuable for studying the factors mediating such post-transcriptional control.

### *Dynamic association of functionally related RBPs within the 3ʹUTR of* p27 *mRNA*

**Figure 3.**
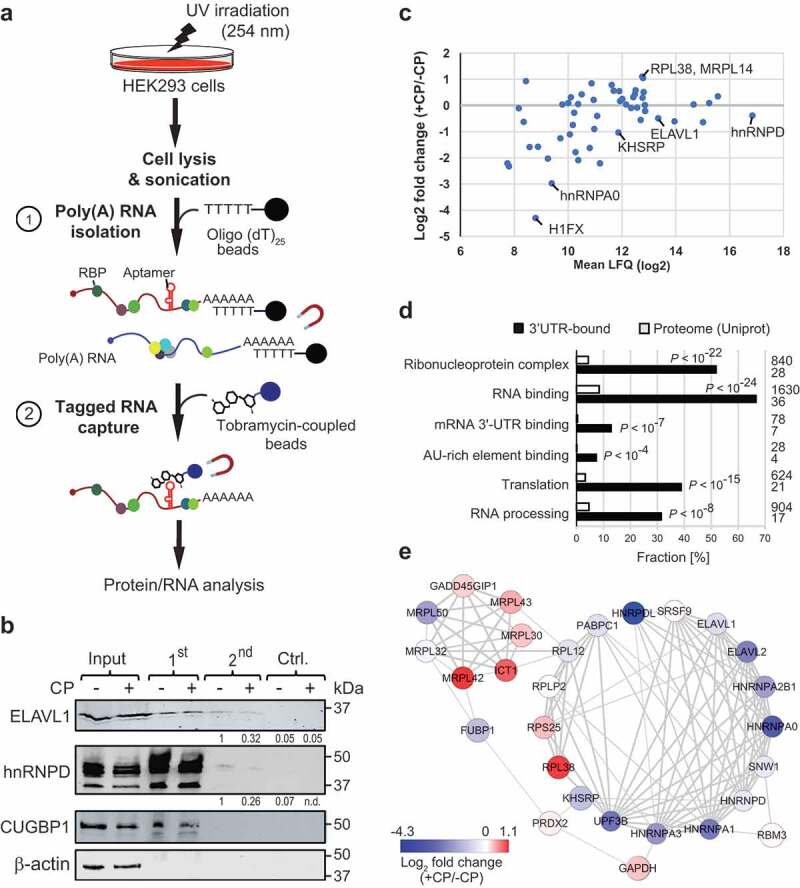
TobTRIP and MS analysis identifies a network of functionally related RBPs bound to p27(3ʹUTR) reporter mRNAs. (a) Schematic representation of tobTRIP for isolation of tagged mRNAs together with bound proteins: In a first step, poly(A) RNA-protein complexes are isolated using oligo(dT)_25_ beads. In a second step, tobramycin aptamer-tagged RNAs are captured with tobramycin-coupled magnetic beads. (b) Immunoblot analysis of the indicated proteins across different steps of tobTRIP. 1:600 of the extract (input), and 1:60 of the 1^st^ and 2^nd^ step eluates were loaded. A quantification of band intensities across the 2^nd^ step eluates is indicated for ELAV1 and hnRNPD. (c) Differential binding of the 54 proteins interacting with GFP-T-p27(3ʹUTR) in CP-treated (+CP) versus untreated (-CP) cells. Mean LFQ values are plotted against log_2_-transformed average foldchanges obtained from normalized intensities. (d) GO terms significantly enriched among the 54 selected proteins. Bars indicate the fractions of proteins annotated with the respective GO term, either across proteins pulled-down with the p27(3ʹUTR) reporter (black bars) or across the entire reference proteome (Uniprot; 20,395 proteins; white bars). Absolute numbers of proteins are shown to the right. Adjusted *P*-values (Benjamini-Hochberg) for enrichments relative to the reference proteome are indicated. (e) PPI network of 28 proteins with multiple and propagating interactions. Each node corresponds to one RBP, and the physical interaction between RBPs is shown as an edge. The thickness of edges is proportional to the STRING confidence score (between 0–1). Node colour corresponds to average log_2_ fold-change of RNA binding in treated (+CP) versus untreated (-CP) cells as indicated in the colour bar.

To identify proteins interacting with the 3ʹUTR of *p27*, we established tobTRIP to capture *in vivo* formed RNA-protein complexes (Figure 3(a)). After UV irradiation of HEK293 GFP-T-p27(3ʹUTR) cells to crosslink proteins to RNA *in vivo*, polyadenylated (poly(A)) RNAs were captured from a cell lysate using oligo(dT)_25_ beads [38,39]. In a second step, the tagged mRNAs were enriched with tobramycin-coupled magnetic beads (see Materials and Methods). To control for non-specifically enriched mRNAs and proteins, the second purification step was performed with beads devoid of tobramycin. This control facilitates the monitoring of unspecific binding to beads using the same input sample; however, it does not exclude the possibility for enriching crosslinked RNA-protein complexes that may directly interact with tobramycin. In total, we performed three independent isolations (biological replicates) from CP-treated and untreated HEK293 cells with corresponding control samples (12 samples in total).

The recovery of tagged mRNAs was monitored by RT-qPCR, revealing selective enrichment of *GFP* mRNA. Nevertheless, we also observed some enrichment in respective control samples (beads devoid of tobramycin), possibly reflecting non-specific binding of mRNAs to beads (Supplemental Figure S5). The presence of RBPs in the affinity isolates was further examined by immunoblot analysis. ELAVL1 and hnRNPD, both known to interact with the 3ʹUTR of *p27* mRNA [40–42], were both detected in tobramycin affinity isolates but not in control samples (Figure 3(b)). Conversely, CUGBP1, an RBP reported to bind to the 5ʹUTR of *p27* [43] was identified in poly(A) RNA isolates (1^st^ step) but was not detected in tobramycin eluates. β-actin was not detectable in either isolate.

To identify mRNA-bound proteins, we subjected all samples to LC-MS/MS and processed and analysed the data with MaxQuant LFQ mode [44]. Three hundred and fifty-five proteins were detected with a false-discovery rate (FDR) of less than 1%. After removing reverse peptides and contaminants 102 proteins were identified by at least two different peptides in at least two out of six samples. For further analysis, we considered only the 54 proteins that were at least 2.5-fold enriched above the negative control samples (Figure 3(c); a list of the 54 selected proteins and the complete MaxQuant analysis with LFQ and less-stringent iBAC analysis mode for comparison is given in the Supplemental Dataset S1).

Gene Ontology (GO) enrichment analysis revealed a significant enrichment of RBPs among the 54 proteins (36 proteins annotated to GO-term ‘RNA binding’, *P* < 10^−24^), primarily acting either in ‘RNA processing’ (*P* < 10^−8^) and/or translation (*P* < 10^−15^) (Figure 3(d); an extended subset of significantly enriched GO terms is provided in Supplemental Table S1). Manual inspection also revealed several proteins that have functional links to DNA damage response and apoptosis (e.g., CTNNB1, DSG1, DSP, HMGB1, HMGB2, DSC1, JUP, LTF, S100A8, PDAP1, CASP14, SAFB2); and six proteins (KHSRP, hnRNPA1, PABC1, PRDX2, NPM1 and GAPDH) have been directly linked to the CP response in human as recorded in the Comparative Toxicogenomics Database (CTD [45]), which is more than expected by chance (*P* = 0.001, hypergeometric distribution; total 702 human proteins linked to CP in CTD, representing 2.2% of all 30,585 human protein genes in CTD). Thus, besides the expected overrepresentation of RBPs, the identified proteins instil functional annotations that relate to the chosen cellular stress conditions.

Nevertheless, CP treatment induced only modest changes of the associations of mRNA-bound proteins (Figure 3(c)). Two RBPs showed more than a two-fold increase in associations with the reporter mRNA in CP-treated cells, including RPL38, a ribosomal protein reported to particularly regulate the translation of *HOX* genes [46], while MRPL14 codes for a mitochondrial ribosomal protein that, we speculate, may relocate from mitochondria in response to CP-induced mitochondrial membrane permeabilization [15]. Conversely, 14 proteins (25% of the 54 selected proteins) were at least two-fold less associated with *p27(3ʹUTR)* reporter mRNAs in CP-treated cells. This includes 11 known RBPs (KHSRP, ELAVL2, FUBP1, UPF3B, hnRNPs A0, A1, A2B1, and A3; and the nucleolar proteins nucleolin and NOLC1) as well as the DNA-binding proteins SUB1, a transcriptional co-activator, and H1FX, a histone protein identified as poly(A) RNA-binding protein in previous RNA-protein interactome studies [47]. Of note, other RBPs such as ELAVL1 (log_2_ fold-change = −0.60) and hnRNPD (log_2_ fold change = −0.39) showed also slightly reduced mRNA associations, broadly recapitulating the changes observed in the immunoblot analysis (Figure 3(b)).

Interestingly, protein–protein interaction (PPI) network analysis with STRING revealed more interactions among interacting proteins than would be expected by chance (total 53 nodes/proteins, 111 edges, *P* < 10^−16^), supporting the notion that the captured proteins are biologically connected. Thereby, PPI analysis revealed a highly connected subnetwork of RBPs that preferentially showed reduced binding to the reporter mRNA upon CP treatment (Figure 3(e)). The subnetwork contains RBPs that function in the regulation of mRNA stability and translation, such as ELAVL1, hnRNPD/AUF1, hnRNPA0, and KHSRP (*P* < 10^−4^), which are known to bind to AU-rich elements (AREs) in 3ʹUTRs of mRNAs [48]. Since the 3ʹUTR of *p27* mRNA contains several AREs including six ‘AUUUA’ core motifs [49,50], it seems possible that remodelling of these ARE RBPs upon CP treatment could affect *p27* mRNA stability or translation. Overall, the significant physical and functional associations among interacting proteins indicate a post-transcriptional regulatory network involving ARE-binding and other RBPs that likely control the fate of *p27* mRNA in a combinatorial fashion.

### *Knock-down of candidate RBPs affects upregulation of* p27 *mRNA levels upon CP treatment in HEK293 cells*

To validate our findings, we further focused on five RBPs that were previously identified as a poly(A) RNA interacting proteins [38] and are implicated in cell-cycle control and/or the DNA damage response. The five include ELAVL1 and hnRNPD, both known to interact with AREs in the 3ʹUTR of *p27* mRNAs [40–42]; KHSRP, an ARE-BP that regulates the stability and/or translation of mRNAs coding for proteins with roles in cell proliferation, differentiation and cancer [51]; SNW domain-containing protein 1 (SNW1), which was shown to regulate the transition from G1 to S phase by controlling *cyclin D1* mRNA stability [52]; and platelet-derived growth factor A (PDGFA) associated protein 1 (PDAP1), a mitogen-associated phosphoprotein involved in the DNA damage response and shown to be induced in HeLa cells exposed to 5-fluorouracil and CP [53].

We first determined whether mRNA and protein levels for these RBPs changed in CP-treated HEK293 cells, which could explain the altered associations with reporter *p27(3ʹUTR)* mRNA in CP-treated cells. While ELAVL1, SNW1, PDAP1 protein as well as *ELAVL1* and *SNW1* mRNA levels were slightly increased in CP-treated cells, no significant changes were observed for KHSRP and hnRNPD (Supplemental Figure S6). Thus, the observed slightly diminished interaction for RBPs ELAVL1, hnRNPD, KHSRP, and SNW1 with *p27(3ʹUTR)* reporter mRNAs upon CP treatment does not coincide with – and unlikely dependent on – changes in their abundance. However, the observed slight increased association of PDAP1 (~1.2-fold) with the *p27(3ʹUTR)* reporter mRNA in CP-treated cells' coincidences with the substantially increased abundance of PDAP1 protein in CP-treated HEK293 cells, which is reminiscent to previous observations made in HeLa cells exposed to CP [53].

**Figure 4.**
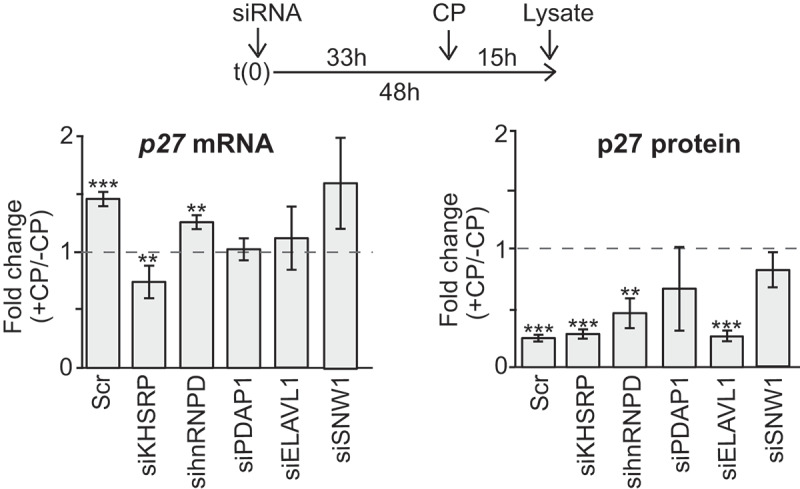
Knock-down of candidate RBPs affect *p27* mRNA abundance upon CP treatment in HEK293 cells. (a) Cells were transiently transfected with siRNAs (siKHSRP, sihnRNPD, siPDAP1, siELAVL1, siSNW1) and Scr control oligos for 48 h and treated with 20 µM CP for the last 15 h. *P27* mRNA levels of CP-treated (+CP) relative to untreated cells (-CP) was assessed by RT-qPCR and normalized to *β-actin* (left). P27 protein levels were quantified with immunoblots (right). Error bars represent SEM, n = 3. *P < 0.05, **P < 0.01, ***P < 0.001, two-tailed student’s t-test.

To further investigate the role of the RBPs in regulating *p27* mRNA levels in the presence of CP, we knocked-down each of the five selected RBPs with siRNAs and measured changes of p27 mRNA and protein levels upon CP treatment of cells (Figure 4). The previously seen induction of *p27* mRNA levels upon CP treatment was recapitulated with scrambled (Scr) control siRNA treated HEK293 cells, indicating that siRNA transfections do not greatly affect the outcome. We observed an induction of *p27* mRNA levels upon CP treatment in hnRNPD and SNW1 knock-down cells, while the knock-down of PDAP1 and ELAVL1 attenuated the induction of *p27* mRNA. Of note, similar results were obtained for ELAVL1 knock-downs upon normalization of RT-qPCR data to *tubulin* (*TUBB*, 1.23-fold) or *GAPDH* mRNAs (0.85 fold) as compared to *β-actin* mRNA which was reported to interact with ELAVL1 [54]. Most striking, knock-down of KHSRP led to a significant decrease of *p27* mRNA levels in CP-treated cells. These results suggest that KHSRP, and possibly ELVAL1 and PDAP1, are implicated in the CP-mediated induction of *p27* mRNA. However, since the efficiency of RPB knock-downs varied between experiments and RBPs, the absence of measurable effects on p27 levels should be interpreted with some caution, especially for SNW1 and PDAP1 (representative Western blots and knock-down of RBPs are shown in the Supplemental Figure S7). We also monitored p27 protein levels, which we previously observed to slightly decrease upon CP treatment of HEK293 cells (Figure 1(d)). siRNA controls as well as knock-down of KHSRP, hnRNPD and ELAVL1 led to similar reductions of p27 protein levels upon CP treatment, whereas it was less pronounced in PDAP1 and SNW1 knock-downs (Figure 4; Supplemental Figure S7). Therefore, as previously observed, the changes in p27 mRNA levels upon CP treatment are not necessarily correlated with the changes of protein levels, which could either relate to additional regulatory events, such as protein degradation or modification, or functions of the mRNA apart from being a template for translation.

### KHSRP modulates CP-induced alterations of p27 mRNA levels and renders MCF7 cells sensitive to CP treatment

**Figure 5.**
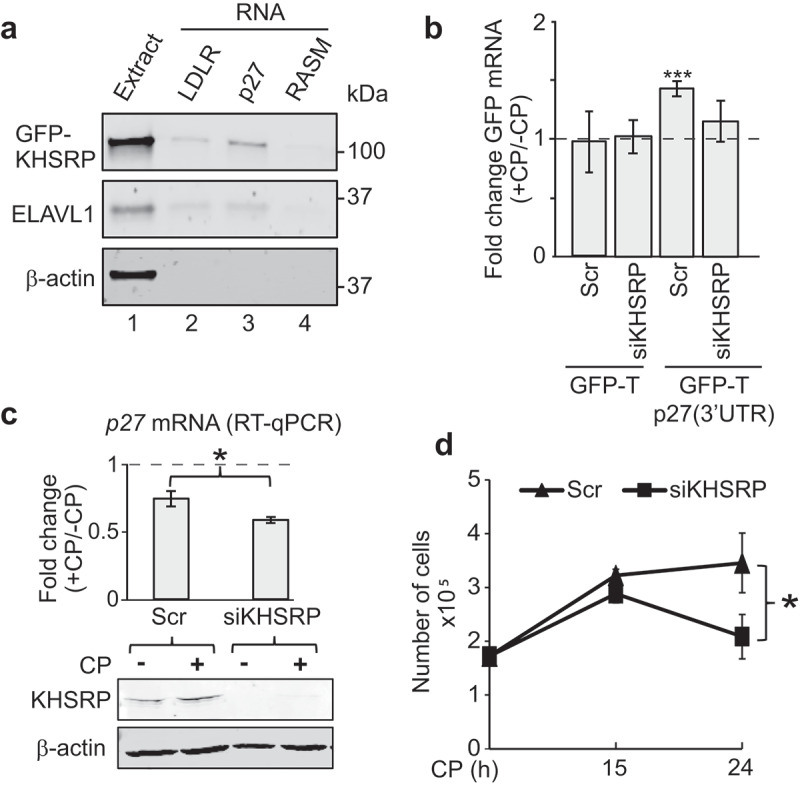
KHSRP affects *p27* mRNA abundance via the 3ʹUTR and modulates CP sensitivity of MCF7 cancer cells. (a) Extracts prepared from HEK293 cells expressing GFP-tagged KHSRP (lane 1) were incubated with biotinylated RNAs comprising a fragment containing AREs of the 3ʹUTR of *LDRL* mRNAs (lane 2), the 3ʹUTR of *p27* mRNA (lane 3), and *RASM* as a negative control (lane 4). RNA was captured with streptavidin beads and monitored for the presence of GFP-KHSRP, ELAVL1 and actin by immunoblot analysis with GFP, ELAVL1, and actin antibodies, respectively. (b) HEK293 cells expressing GFP-T and GFP-T-p27(3ʹUTR) were transiently transfected with siKHSRP or scr (siRNA control) for 48 h and treated with 20 µM CP for the last 15 h. The level of GFP in CP-treated (+CP) *versus* untreated cells (-CP) was assessed by RT-qPCR normalized to *β-actin*. (c) MCF7 cells were transiently transfected with siRNAs targeting KHSRP (siKHSRP) and Scr control oligos. *P27* mRNA levels of CP-treated (+CP, 24 h) relative to untreated cells (-CP) was assessed by RT-qPCR normalized to *β-actin*. An immunoblot showing knock-down of KHSRP is depicted below. (d) Cell proliferation of MCF7 cells was determined by Trypan Blue assay at the indicated time points after CP treatment. Error bars represent SEM, n = 3. *P < 0.05, ***P < 0. 001, two-tailed student's t-test.

Since knock-down of KHSRP led to significantly reduced endogenous *p27* mRNA levels upon CP treatment, we further focused our studies on this RBP that contains three hnRNPK-homology (KH) RNA-binding domains interacting with AREs in the 3ʹUTR of mRNA targets, thereby regulating mRNA stability and translation [51]. To confirm interaction of KHSRP with the *p27* 3ʹUTR, we first performed RNA pull-down experiments using synthetic biotinylated RNAs added to extract derived from cells expressing GFP-tagged KHSRP (Figure 5(a)). GFP-KHSRP selectively interacted with the 3ʹUTR of *p27* and an RNA fragment derived from the *LDLR* 3ʹUTRs, a previously validated target bearing three AREs [55]. Similar interactions were detected with ELAVL1. In either case, no interaction with an unrelated control RNA (*RASM*) was observed. Of note, we also tested a predicted KHSRP binding site in the 3ʹUTR of *p27* (CCUCCC; identified with ScanForMotifs [49]), but we were unable to determine any interaction with this motif in our assay (data not shown).

We next monitored GFP-T-p27(3ʹUTR) and GFP-T levels in KHSRP siRNA knocked-down cells to evaluate whether changes in *p27* mRNA abundance by KHSRP following CP treatment were mediated via the 3ʹUTR (Figure 5(b)). No induction was seen in GFP-T control cells transfected with siRNA controls or siRNA against KHSRP upon treatment with CP. In contrast, an induction of GFP-T-p27(3ʹUTR) following CP treatment was recapitulated in siRNA control transfections, but it was significantly alleviated upon siRNA mediated knock-down of KHSRP. These data suggest that KHSRP is critically involved in the post-transcriptional regulation of *p27* mRNA levels upon CP treatment through interaction with 3ʹUTR sequences.

Finally, we wondered whether KHSRP may also influence *p27* mRNA levels in response to CP treatment in MCF7 cells. Knock-down of KHSRP in MCF7 cells resulted in slightly, but significantly, reduced mRNA levels of *p27* upon CP treatment, which is in line with our previous observations made in HEK293 cells (Figure 5(c)). Interestingly, while cell-proliferation was generally not affected by siRNA mediated knock-down of KHSRP, we observed that it significantly attenuated proliferation after 24 h of CP treatment as compared to control siRNA transfected cells (Figure 5(d)). These results suggest that KHSRP is part of a regulatory network that controls *p27* mRNA levels, and it is potentially involved in mediating CP response and the inferred sensitivity of cancer cells to the drug. Nonetheless, whether *p27* mRNA regulation is directly linked to CP response possibly involving KHSRP and other RPBs will need to further investigation.

## Discussion

Drug resistance in cancer treatments is a worldwide problem. While diverse factors contribute to CP resistance [14], the implications of post-transcriptional events in drug sensitivity are just starting to be uncovered [56,57]. In this study, we observed different fates of *p27* mRNA upon 15 h CP treatment of HEK293 compared to MCF7 breast cancer cells. Focusing on CP-sensitive HEK293 cells, we found that *p27* mRNA was stabilized upon CP treatment and that the 3ʹUTR contributes to mRNA stabilization. To monitor interacting RBPs we developed tobTRIP, a novel biochemical approach for capturing *in vivo* formed RNPs on affinity-tagged 3ʹUTRs. This allowed us to identify 54 proteins that reproducibly interacted with the tagged *p27(3ʹUTR)* mRNAs. About 25% of these RBPs exhibited more than a two-fold change in their level of *p27* mRNA associations upon CP treatment, including KHSRP and other ARE-binding RBPs that likely form a post-transcriptional regulatory network. Knock-down of selected RBPs impeded CP induction of *p27* mRNA levels in HEK293 cells, further suggesting a regulatory function. Moreover, likely obstruction of the post-transcriptional regulatory network in MCF7 breast cancer cells by knock-down of KHSRP significantly reduced cell proliferation upon CP treatment, emphasizing the role of RBPs in cancer biology and drug response.

One apparently paradoxical observation concerned the stabilization of *p27* mRNAs upon CP treatment of HEK293 cells, while translation was generally reduced. This seems counterintuitive as one may expect that stabilization of mRNAs would go along with increased translation or the storage of untranslated mRNAs (i.e., in stress granules). However, mRNAs could also have other regulatory functions in cells, albeit little is known about potential ‘non-coding’ functions of mRNAs. For instance, mRNAs or particular regions thereof (e.g., UTRs) can act as scaffold for protein complex assembly [58], anneal with other RNAs analogous to endogenous competing RNAs (ceRNA) that sequester miRNAs and thereby diminish their interaction with other mRNAs [59]; or they could be involved in transcriptional control and epigenetic functions [36]. Further investigations will be required to test these possibilities.

At this point, our studies were particularly focused to develop tobTRIP, an approach aimed to reveal interactions of proteins with 3ʹUTRs of specific mRNA *in vivo* and taking the post-transcriptional control of *p27* mRNA in CP-treated cells as a model system for investigation. In brief, tobTRIP is based on a cell-integrated reporter mRNA comprised of the coding sequences for eGFP, a tobramycin RNA aptamer sequence for affinity purification (HAMMER2 cassette) followed by the 3ʹUTR sequence under study. Since all components of the reporter system can be exchanged by cloning via restrictions sites, our primary set-up can be modified and expanded to other UTRs or RNA aptamers, allowing streamlined applications for parallel testing of many conditions. For instance, we found that the tobramycin aptamer was superior to other widely used aptamers such as the streptomycin S1 aptamer [60], as we experienced that recovery of S1-tagged RNAs was less efficient, which is possibly due to its lower affinity for streptavidin (unpublished observations). In this regard, we also observed that initial enrichment of poly(A) RNAs with oligo(dT) beads from extracts was beneficial for selection affinity-tagged RNAs in a second step, as we experienced that direct capture of cross-linked RNP complexes from extracts was not efficient and led to higher background. Finally, since the mRNA reporter expresses GFP, it can also be used to compare the influence of different 3ʹUTRs on expression and localization *in vivo* e.g., for validation of interaction partners.

Our analysis of the proteins bound to tagged *p27(3ʹUTR)* RNA revealed enrichment of an array of RBPs, some of them previously linked to *p27* mRNA regulation. This included ELAVL1, hnRNPD as well as hnRNPA0 enforcing cell-cycle checkpoints, allowing DNA repair and tolerance to chemotherapy [24]. However, we note that we could not detect all previously reported RBPs that regulate *p27* mRNA through interaction with 3ʹUTR sequences (e.g., PUMILIO proteins [61]). The reason for this lack of detection could be manifold, ranging from limited MS sensitivity, low expression and biological variation in binding affinities across different cell-types. Nevertheless, our analysis revealed a subnetwork of functionally connected RBPs that most likely combinatorially control the fate of *p27* mRNAs. Besides sharing physical network relations, the 54 selected proteins also have significant functional relations, such as the CP response, as revealed by data extraction from the CTD. Thus, while RBPs often bind to mRNAs coding for functionally related proteins, forming so-called RNA regulons [62,63], we speculate that the reverse may apply in certain instances: proteins interacting with certain parts of an mRNA may establish a functional coherent protein assemblage with potential for predicting functional outcomes.

SiRNA-mediated knock-down of three (KHSRP, ELVAL1 and PDAP1) of the five selected interacting proteins revealed significant alterations on CP-mediated induction of *p27* mRNA levels in HEK293 cells. Thereby, most striking effects were observed in knock-downs of KHSRP, whose association and imposed regulation on 3ʹUTR sequences of *p27* was confirmed by *in vitro* RNA pull-down assays and reporter assays, respectively (Figure 5(a), (b)). Interestingly, knock-down of KHSRP rendered MCF7 cells more sensitive to CP treatment (Figure 5(d)). Although it is not yet resolved whether this increased sensitivity is directly linked to altered *p27* mRNA levels, it is in line with previous observations that suggest the involvement of KHSRP in DNA damage responses induced by related drugs, such as doxorubicin [64], bleomycin [65], and etoposide [66]. Furthermore, alterations of KHSRP levels have been reported upon CP treatment in different cell-lines [67,68]. Interestingly, phospho-proteome profiling in stem cells indicated that KHSRP is phosphorylated after 4 h of CP treatment [19]. Since CP-treatment leads to activation of the p38/MK2 signalling pathway [69] and p38/MK2 can phosphorylate threonine 692 in the C-terminal domain of KHSRP, which negatively regulates ARE-binding capabilities [70], it is tempting to speculate that phosphorylation of KHSRP through p38/MK2 could lead to the observed reduced mRNA binding in CP-treated HEK293 cells. Although this hypothesis needs further testing, a recent comparative RNA-protein interactome study in mouse fibroblasts subjected to DNA stress (etoposide) has highlighted the role for activation of the p38/MK2 signalling pathway in the regulation of cell cycle progression [66]. In agreement with our study, it disclosed a cluster of RBPs including KHSRP and other RBPs (i.e., ELAVL1, hnRNPDL, hnRNPA0, and PABPC1) identified in our study that showed reduced mRNA associations upon etoposide treatment [66].

In conclusion, our biochemical approach for characterization of RBPs interacting with 3ʹUTRs highlights critical factors for the regulation of *p27* mRNAs upon CP treatment. It revealed that KHSRP is likely part of a post-transcriptional regulatory network that involves several ARE-BPs, possibly establishing a new component for modulation of CP drug sensitivity in cancer cells. While the potential link between *p27* mRNA regulation and CP response needs to be further established, our conceptual approach could be used to investigate the impact of mutations in the often-disregarded regions of transcripts, such as UTRs, which should be taken into consideration in the future era of pharmacogenomics and personalized medicine.

## Materials and methods

### Cell culture and proliferation assays

Michigan Cancer Foundation-7 breast adenocarcinoma (MCF7) cells were purchased from the European Collection of Authenticated Cell Cultures (ECACC, #86,012,803), Human Embryonic Kidney 293 (HEK293) and Flp-In-293 cells were obtained from Invitrogen (#R750-07). Cells were grown in Dulbecco’s Modified Eagle Medium (DMEM) with High-GlutaMax-I (Life Technologies, #31,966-021) supplemented with 100 U/ml penicillin, 100 μg/ml streptomycin (Sigma, #P4333), and 10% foetal bovine serum (FBS; Sigma, #F7524) in standard tissue culture dishes in a humidified incubator at 37°C and 5% CO_2_.

*Proliferation assays*: HEK293 and MCF7 cells were seeded (5 × 10^5^ cells in each well) in triplicate in 12-well cell-culture dishes, treated with 20 µM of cisplatin (Sigma, #C2210000) or untreated as control and further grown for the indicated times (from 0 to 48 h). Cells were washed twice with 500 µl of phosphate-buffered saline (PBS), trypsinized and re-suspended in 500 µl PBS and counted with a haemocytometer. The fraction of live versus dead cells was determined with Trypan Blue: 10 µl of cell suspension was mixed with 10 µl Trypan Blue (Life Technologies, #T10282), loaded on a disposable slide (Invitrogen, #C10283) and counted with a Countess™ II FL Automated Cell Counter.

### Plasmid construction

The DNA sequences comprising the tobramycin RNA aptamer, flanking sequences and multicloning sites (MCS), which we refer to as HAMMER2, were synthesized by Eurofins (MWG, Operon) and provided in pEX-A2 vectors. A fragment containing HAMMER2 was subcloned via flanking *BamH* I and *Xho* I restriction sites into pcDNA-5/FRT/TO-based expression vectors (Invitrogen) that contain a tet-inducible CMV promotor, generating plasmid pTO-HAMMER2. The coding sequence of eGFP was amplified from pTO-HA-Strep-GW-FRT-eGFP (kindly provided by Alexander Wepf, ETH Zurich) with primers *Hind*III-Kozak-eGFP-fwd and eGFP-rev-MCS-*Bam*HI (a list of oligonucleotides used in this study is provided in Supplemental Table S2). After verifying correct insertion by sequencing, the eGFP cassette was subcloned into the respective pTO derivative via *Hind* III and *Bam*HI, generating plasmid pTO-GFP-T. The 3ʹUTR of CDKN1B/p27 (nts 1,070–2,403; RefSeq: NM_004064.3) was amplified by PCR from pGL3-CDKN1B-3ʹUTR (kindly provided by Dr. Martijn Kedde, NKI Amsterdam), with primers CDKN1B-3ʹUTR-fwd and CDKN1B-3ʹUTR-rev that contain *Xho* I and *Not* I restriction sites, respectively. The PCR fragment was then inserted into pCR-Blunt II-TOPO (Invitrogen) generating plasmid pCR-Blunt II-TOPO-p27(3ʹUTR). After sequencing, the fragment bearing the CDKN1B/p27 3ʹUTR was excised and subcloned via *Xho* I and *Not* I restriction sites to generate pTO-GFP-T-p27(3ʹUTR).

### Generation of stable cell lines and transfections

Tet-inducible stable clones were generated in Flp-In-293 cells (Invitrogen, #R750-07) by co-transfection with either 10 µg of pTO-GFP-T or pTO-GFP-T-p27(3ʹUTR) plasmids and 1 µg of plasmid pOG44 with Lipofectamine 2000 (Invitrogen, #11,668-027). Stable clones were selected in the presence of 200 µg/ml of hygromycin B (Invitrogen, #10,697-010) and maintained in 50 µg/ml hygromycin B. GFP-T and GFP-T-p27(3ʹUTR) HEK293 cells were cultured in 12-well plates and transfected with small-interfering RNAs (siRNAs) and control siRNAs (Scr [71]) at ~60% cell confluency using Lipofectamin RNAiMAX reagent (Life Technologies, #13,778-100). In brief, 3 µl of Lipofectamine RNAiMAX was diluted in 50 µl of Opti-Mem (Gibco, # 31,985,070) and combined with 10 pmol of respective siRNA supplied in 50 µl of Opti-Mem and incubated for 15 min at room temperature (RT). The mixture was added to cells, which were further grown in media supplemented with 1 µg/ml of tet (Fisher, #BP-912-100) and treated with 20 µM of CP for 15 h. Plasmid transfections were performed at 70% cell confluency with 2 µg of pEGFPC1-6XHis-FLKSRP (Addgene, #23,001) and Lipofectamine 2000 (Invitrogen, #11,668-027).

### Polysome gradient fractionation

Cell lysis and polysomal fractionation were essentially performed as described [72]. In brief, GFP-T-p27(3ʹUTR) cells were lysed in the dish with 300 µl of lysis buffer (10 mM Tris-HCl, pH 7.5, 10 mM NaCl, 10 mM MgCl2, 1% Triton X-100, 1% sodium deoxycholate, 1 mM dithiothreitol (DTT), 40 U/mL RNasin (Promega, #N2611)). Cytoplasmic extracts were loaded onto a 20–50% linear sucrose gradient containing 30 mM Tris–HCl (pH 7.5), 100 mM NaCl and 10 mM MgCl_2_. Gradients were centrifuged in a Beckman SW 41 rotor for 2 h at 37,000 r.p.m. and collected in 12 fractions while continuously monitoring the absorbance at 254 nm. A control RNA (LysA) was added to each fraction prior to RNA isolation with Trizol and isopropanol precipitation [73].

### Fluorescence microscopy

*Live cell imaging*: HEK293 cells with GFP-T or GFP-T-p27(3ʹUTR) were grown on a cover slip and GFP expression was induced with 1 µg/ml tet for 48 h. Cells were visualized under phase contrast and fluorescence microscopy was performed with the GFP filter on an EVOS FL Cell Imaging System (ThermoFisher Scientific).

*Immunofluorescence of fixed cells*: Stress granule (SG) formation was induced with 0.5 mM sodium (meta) arsenite (NaAsO_2_; Sigma, #S7400) for 15 min. Cells were washed gently with PBS and immediately incubated in 1 ml of fixing solution (4% formaldehyde in PBS) for 10 min, and further permeabilised with 1 ml of 0.1% Triton X-100 in PBS for 15 min at RT. Blocking was carried out with 1 ml of blocking solution (1% BSA in PBS) for 1 h at RT. Fixed cells were then incubated with primary Ras GTPase-activating protein-binding protein 1 (G3BP1; BD Biosciences, #611,127) antibodies diluted 1:50 in PBS for 2 h at RT, and washed three times with PBS prior to the addition of anti-mouse secondary antibodies (1:200; Jackson ImmunoResearch, #715-025-150) for 1 h at RT in the dark. Finally, cells were washed three times with PBS and mounted on the slide with ProLong Gold antifade with 4′,6-diamidino-2-phenylindole (DAPI; Life Technologies, #P36935). Confocal images were acquired on a Nikon Ti-Eclipse A1M microscope fitted with a 60× oil immersion objective using 488 nm, 561 nm and 405 nm laser excitation lines.

### Immunoblot analysis

Proteins were resolved on SDS polyacrylamide (PAA) gels (12.5%) and transferred to nitrocellulose membranes (Bio-Rad, #1,620,115). Membranes were blocked in PBS containing 0.1% Tween-20 and 3% BSA and probed with the designated primary antibodies and with IRDye 800CW (Licor #926-32,210, #926-32,211) or IRDye 650RD secondary antibodies (Licor #926-68,070, #926-68,071). The blots were visualized with the Odyssey® CLx Imaging System. The following primary antibodies were used: anti-β-actin (1:2,000; Sigma, #A1978), anti-β-Tubulin (1:2,000; Sigma, #T0198), anti–ELAVL1 (1:500; Santa Cruz, #sc-5261), anti-KHSRP (1;1000; Cell Signalling, #13,398), anti-SNW1 (1:1,000; Abcam, #ab67165), anti-PDAP1 (1:500; Cell Signalling, #4300), anti-p53 (1:1,000; Cell Signalling, #9282), anti-CDKN1B/p27 (1:500; Cell Signalling, #3686), anti-hnRNPD (1:1,000; Millipore, #07-260), anti-CUGBP1 (1:500; Santa Cruz, #sc-20,003) and anti-GFP (1:2,000; Roche, #11,814,460,001).

### UV crosslinking of cells and extract preparation

Three 150 × 20 mm tissue culture dishes (Corning, #430,599) of GFP-T-p27(3ʹUTR) HEK293 cells (total 6 × 10^7^ cells) were treated with 1 µg/ml of tet for 48 h before harvesting. To induce the cisplatin response, 20 µM of CP was added to tet-treated cells after 33 h for 15 h. Thereafter, cells were washed twice with 10 ml pre-warmed PBS and after removal of the final rinse, 6 ml of PBS was added. Cells were exposed to UV light for crosslinking of protein-RNA complexes *in vivo* and cell-free extracts were prepared as described previously [10]. In brief, cells were exposed on ice to UV light (254 nm) at 150 mJ/cm^2^ in a Stratalinker 1800 (Stratagene) and centrifuged at 250 g for 5 min at 4ºC. The cell pellet was resuspended in 2 ml of lysis buffer (100 mM Tris-HCl, pH 7.5, 500 mM LiCl, 10 mM EDTA, 1% Triton X-100, 5 mM DTT, 20 U/ml DNase I (Promega, #M6101), 100 U/ml RNasin (Promega, #N2611), complete EDTA-free protease-inhibitor cocktail (Roche, #11,836,170,001)). Cell lysates were then combined (total ~6 ml) and subjected to three rounds of sonication (Soniprep150, MSE) and cleared by centrifugation at 15,000 g for 10 min at 4°C. Protein concentrations of extracts were determined using the Bradford assay with BSA as a reference standard.

### Tobramycin-based tandem RNA affinity isolations

*1^st^ step – isolation of poly (A) RNAs*: Poly(A) RNAs were isolated from extracts with the Dynabeads mRNA Purification Kit (Life Technologies, #61,011) essentially as described [10]. Dynabeads (1.2 ml) were equilibrated twice with 3 ml of lysis buffer and then combined with 5.5 ml of cell extract (~50 mg protein) and further incubated for 10 min at 25°C upon continuous shaking. The beads were collected with a magnet and washed with buffer A (10 mM Tris-HCl, pH 7.5, 300 mM LiCl, 1 mM EDTA, 0.1% Triton X-100) and twice with buffer B (10 mM Tris-HCl, pH 7.5, 150 mM LiCl, 1 mM EDTA) and finally re-suspended in 300 µl of 10 mM Tris-HCl, pH 7.5. Poly(A) RNA was eluted at 80°C for 2 min. The entire procedure was repeated three times by reapplying the supernatant to oligo(dT)_25_ beads.

*2^nd^ step – affinity isolation of GFP-T-p27(3ʹUTR) mRNAs*: GFP-T-p27(3ʹUTR) RNA was captured using the RNA aptamer for tobramycin, essentially following the strategy for purification of ribonucleoprotein (RNP) complexes [74]. 100 mM tobramycin (Sigma, #T1783) was freshly prepared in coupling buffer (0.2 M NaHCO_3_, pH 8.3, 0.5 M NaCl). The NHS activated magnetic beads (Pierce, #88,826) were equilibrated at RT, mixed thoroughly for 10 s on a vortex, and 300 µl of beads were placed in a 1.5 ml protein LoBind tube (Sigma, #Z666505). The beads were collected using a magnetic stand and washed by gently mixing for 15 s in 1 ml of ice-cold 1 mM hydrochloric acid. After collecting the beads, 1 ml of 5 mM tobramycin provided in coupling buffer was added and the mixture was incubated over night at 4°C with shaking. The beads were collected and washed twice with 1 ml of 0.1 M glycine (pH 2.0) for 15 s and washed once with 1 ml of ultrapure water (Sigma). Then, the beads were incubated with 1 ml quenching buffer (3 M ethanolamine-HCl, pH 9) for 2 h at RT on a rotator and subsequently washed once with 1 ml of ultrapure water and twice with 1 ml of coupling buffer. Beads were kept in 300 µl of coupling buffer supplemented with 0.05% sodium azide at 4°C.

For affinity purification of tagged RNAs, 150 µl of tobramycin-coated beads were blocked in 1 ml of blocking solution (20 mM Tris-HCl, pH 8.1, 300 mM KCl, 1 mM CaCl_2_, 1 mM MgCl_2_, 0.2 mM DTT, 0.1 mg/ml tRNA (from *E. coli* MRE 600, Sigma #000000010109541001), 0.5 mg/ml BSA, 0.01% of Nonidet-P40) overnight at 4°C under constant agitation and then washed twice with RNA-binding buffer (20 mM Tris-HCl, pH 8.1, 145 mM KCl, 0.1 mg/ml tRNA, 1 mM CaCl_2_, 1 mM MgCl_2_, 0.2 mM DTT). Eight hundred microliters of the eluate obtained from the first step (~70 µg poly(A) mRNA from 6 × 10^7^ cells) was adjusted to 900 µl in RNA-binding buffer and combined with the beads and incubated for 2 h at RT under constant shaking. Then, the beads were collected using a magnetic stand and the supernatant saved for further analysis. The beads were further washed six times with 500 µl of buffer C (20 mM Tris-HCl, pH 8.1, 125 mM KCl, 1 mM CaCl_2_, 1 mM MgCl_2_, 0.2 mM DTT, 0.1% NP40). RNA and bound proteins were eluted in 300 µl of elution buffer (20 mM Tris-HCl, pH 8.1, 145 mM KCl, 5 mM tobramycin, 2 mM MgCl_2_, 1 mM CaCl_2_, 0.2 mM DTT) at 50°C for 10 min in a Thermoshaker at 1,000 r.p.m. 250 µl of the final eluate was concentrated using a speed-vac and used for mass spectrometry (MS) analysis. Fifty-microliters were kept for Western blot and reverse-transcription quantitative PCR (RT-qPCR) validation.

### Orbitrap mass spectrometry

Samples were run on an SDS-PAGE gel and each gel lane cut as a single. Each slice was subjected to in-gel tryptic digestion using a DigestPro automated digestion unit (Intavis Ltd.) to minimize manual handling. The resulting peptides were fractionated using an Ultimate 3000 nanoHPLC system in line with an Orbitrap Fusion Tribrid mass spectrometer (Thermo Scientific). In brief, peptides in 1% (vol/vol) formic acid were injected into an Acclaim PepMap C18 Nano-trap column (Thermo Scientific). After washing with 0.5% (vol/vol) acetonitrile 0.1% (vol/vol) formic acid peptides were resolved on a 250 mm × 75 μm Acclaim PepMap C18 reverse phase analytical column (Thermo Scientific) over a 150 min organic gradient, using 7 gradient segments (1-6% solvent B over 1 min, 6-15% B over 58 min, 15-32% B over 58 min, 32-40% B over 5 min, 40-90% B over 1 min, held at 90% B for 6 min and then reduced to 1% B over 1 min) with a flow rate of 300 nl/min. Solvent A was 0.1% formic acid and Solvent B was aqueous 80% acetonitrile in 0.1% formic acid. Peptides were ionized by nano-electrospray ionization at 2.2 kV using a stainless-steel emitter with an internal diameter of 30 μm (Thermo Scientific) and a capillary temperature of 250°C.

All spectra were acquired using an Orbitrap Fusion Tribrid mass spectrometer controlled by Xcalibur 2.0 software (Thermo Scientific) and operated in data-dependent acquisition mode. FTMS1 spectra were collected at a resolution of 120,000 over a scan range (m/z) of 350–1,550, with an automatic gain control (AGC) target of 400,000 and a max injection time of 100 ms. The data-dependent mode was set to TopSpeed and the most intense ions were selected for MS/MS. Precursors were filtered according to charge state (to include charge states 2–7) and with monoisotopic precursor selection. Previously interrogated precursors were excluded using a dynamic window (40s, ±10ppm). The MS2 precursors were isolated with a quadrupole mass filter set to a width of 1.6 m/z. ITMS2 spectra were collected with an AGC target of 5000, max injection time of 50 ms and HCD collision energy of 35%.

### MS data analysis

All raw data were analysed with MaxQuant software version 1.6.0.16 [75] using the UniProt human database (downloaded 2017/05/23). MS/MS searches were performed with the following parameters: oxidation of methionine and protein N-terminal acetylation as variable modifications; carbamidomethylation as fixed modification; Trypsin/P as the digestion enzyme allowing up to two missed cleavage sites; precursor ion mass tolerances of 20 p.p.m. for the first search (used for nonlinear mass re-calibration) and 4.5 p.p.m. for the main search, and a fragment ion mass tolerance of 20 ppm. For identification, a maximum false-discovery rate (FDR) threshold of 1% was applied separately on protein and peptide levels. ‘Match between the runs’ was activated, as well as ‘Label-free quantification’ (LFQ) (at least two ratio counts were necessary to get an LFQ value). Two or more unique/razor peptides were required for protein identification and a ratio count of two or more for label-free protein quantification in at least one sample. This produced LFQ values for a total of 165 protein groups. MaxQuant generated LFQ intensities were normalized such that at each condition/time point the LFQ intensity values added up to exactly 1,000,000, therefore each protein group value can be regarded as a normalized microshare (performed separately for each sample for all proteins that were present in that sample). After normalization, a pseudocount (PC) of 100 was added and values were subsequently log_2_ transformed for further analysis. The MaxQuant processed data in LFQ and iBAC mode are available in the Supporting Dataset S1. The mass spectrometry proteomics data have also been deposited to the ProteomeXchange Consortium via the PRIDE partner repository [76] with the dataset identifier PXD008498.

### RNA isolation and RT-qPCR

The RNA from affinity isolates was extracted with the ZR RNA MiniPrep Kit (Zymo #R1064). Ten microliters of RNA obtained from tobTRIP eluates or 500 ng of total RNA isolated from extracts (input) were combined with 2.5 μM of oligo(dT)_18_ and 30 μM of random hexamer primers and reverse-transcribed (RT-) for 2 h at 42°C with the cDNA Synthesis Kit according to the manufacturers’ instructions (Primer Design; #RT-nanoScript2-150). Quantitative PCR (qPCR) was performed on Applied Biosystems Quant-Studio 7Flex with PrecisionPLUS MasterMix premixed with SYBRgreen (PrimerDesign; #PrecisionPLUS-R-SY) using specific DNA primers in according to the manufacturer’s procedures. Essentially, the enzyme was activated for 2 min at 95ºC followed by 40 cycles [95ºC for 10 s, 60ºC for 1 min]. The melt-curve was included at the end of the run to ensure specificity of the primers. The comparative Ct method was used to measure the amplification of mRNAs relative to *β–actin* [77].

### RNA stability measurements

HEK293 cells were treated for 15 h with CP, while GFP-T and GFP-T-p27(3ʹUTR) cells were induced with 1 µg/ml of tet for 33 h prior to the addition of CP for 15 h. Then, 2 µg/ml of ActD was added for the indicated times prior to RNA isolation. Five hundred nanograms of total RNA was used for RT-qPCR to measure the level of *GFP, p27* and *c-myc* mRNAs relative to *β–actin* mRNA using respective primers for the *p27* ORF, *GFP, c-myc*, and *β–actin*. The comparative Ct method was used to calculate the mRNA levels; and curves were fitted, and half-life determined with GraphPad Prism using ‘one phase decay equation’.

### Synthesis of biotinylated RNAs and RNA-pull-down

DNA templates for biotin-RNA synthesis were prepared by PCR from either 100 ng of genomic DNA (LDLR, RASM) or 80 ng of pGL3-p27-3ʹUTR plasmid with oligonucleotides bearing a T7 RNA polymerase promoter sequence. In particular, the entire 3ʹUTR of *p27* (1,350 nts) and a fragment (nts 85–947) of the *LDRL* 3ʹUTR encompassing three AU-rich elements (AREs) [55] were amplified with oligonucleotide primer pairs T7-p27_3UTR_Fw/P27_3ʹUTR_END_Rv and LDLR_T7Fw/LDRL_Rev1, respectively (Table S2). A fragment (580 nts) of the CDS of *RASM* was amplified as described previously [78]. Biotinylated RNAs were produced with T7-RNA polymerase and the Biotin RNA Labelling Mix (Roche, #11,685,597,910), as instructed by the manufacturer. Cell-free extracts were prepared in IP buffer (20 mM Tris-HCl, pH 8.0, 100 mM NaCl, 2 mM MgCl_2_, 5% glycerol, 1 mM DTT, 0.1% Triton X-100, 0.1 mg/ml heparin, 0.1 mg/ml tRNA, 1 mM phenylmethylsulfonyl chloride (PMSF), 1 µl 20 U/µl RNase OUT (Promega, #10,777,019) and Complete™ mini EDTA-free protease-inhibitor tablets (Roche, #11,836,170,001)) by mechanical disruption of transfected (pEGFPC1-6XHis-FLKSRP) HEK293 cells with glass beads in a Tissue Lyser (Qiagen; 6 × 30 s; 30 Hz, 4°C). Biotin RNA pull-down experiments were performed essentially as described [78]. Three hundred and fifty micrograms of extract was combined with 10 pmol of biotinylated RNAs and RNA-protein complexes captured with 25 µl of streptavidin M280 Dynabeads® (Invitrogen, #11205D) and resolved on a 4-15% gradient SDS-PAA gel for immunoblot analysis.

### Protein annotation and analysis

The human reference proteome (UP000005640) was downloaded from Uniprot considering Swiss-Prot reviewed entries (20,395 annotated proteins; 20. Sept 2018). GO enrichment analysis was performed with the Generic Gene Ontology (GO) Term Finder using the human reference proteome as a background to calculate Benjamini-Hochberg-corrected *P*-values [79]. The protein–protein interaction (PPI) network of all identified RBPs was retrieved from STRING (vers. 10.5 [80]) with evidence sources restricted to experimental evidence and manually curated databases. For visualization purposes, the STRING network was imported into Cytoscape (version 3.7.1) and arranged in circular layout and further arranged manually. The Comparative Toxicogenomics Database (CTD) [45] was queried for ‘Chemical-Gene interaction’ adding ‘cisplatin’ as the chemical and searching for ‘ANY’ Chemical-gene interaction; further selecting ‘protein’ as the Gene form and restrict the analysis to ‘Homo sapiens’ (Taxonomic ID: 9606) (18.02.2019). Data were retrieved for 702 unique human proteins in Excel format. Statistical overrepresentation (hypergeometric distribution) was calculated based on 30,585 human proteins considered in CTD.
