# Supplementary material for: Tandem RNA isolation reveals functional rearrangement of RNA-binding proteins on CDKN1B/p27Kip1 3’UTRs in cisplatin treated cells
Source: RNA Biol. 2019 Sep 16;17(1):33–46. doi: 10.1080/15476286.2019.1662268 (PMC6948961; doi:10.1080/15476286.2019.1662268)
Supplement: Supplemental Material [file krnb-17-01-1662268-s001.zip › Supplementary information/Suppl. Fig. S1-S7_Iadevaia.pdf]

Supplemental Figures
Iadevaia V. et al.

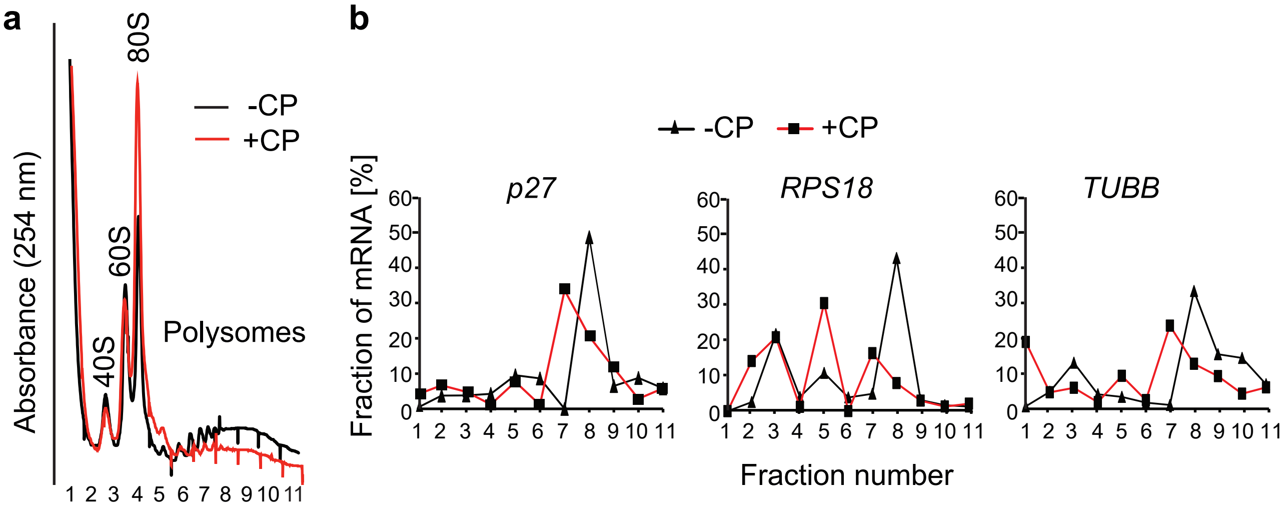

Figure S1. Polysomal profiles of HEK293 cells upon CP treatment. (a) Cytoplasmic extracts were prepared from untreated (-CP) and CP-treated (+CP) HEK293 cells and fractionated on sucrose gradients while continuous monitoring the absorbance at 254 nm. The positions of 40S and 60S ribosomal subunits, 80S monosomes and polysomes are indicated. (b) Distribution of *p27*, *RPS18* and *TUBB* mRNAs in the polysomal gradients. RNA was isolated from each fraction of the polysomal profile and quantified by RT-qPCR. Ct-values were normalised to the LysA control RNA that was added to each fraction of the polysomal gradients (see Materials and Methods). The mRNA level in each fraction was calculated as a percentage of the total.

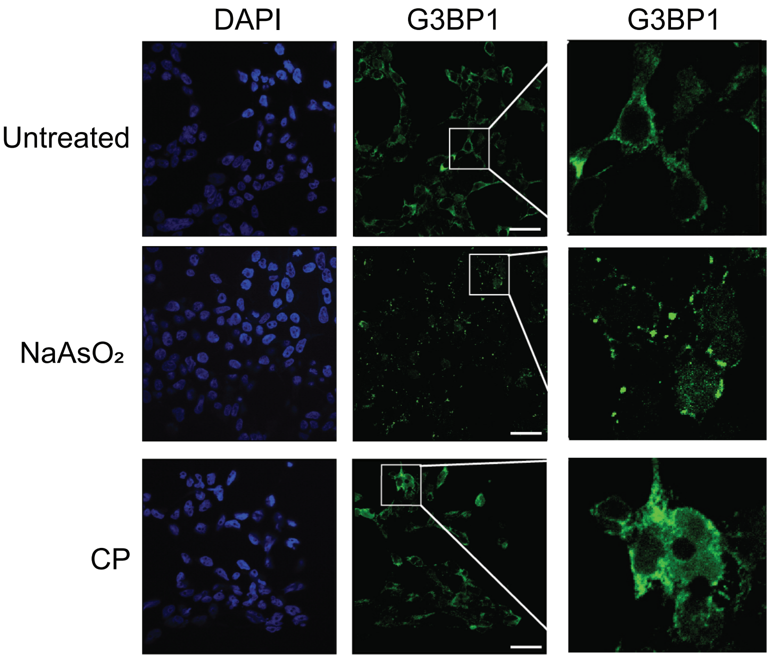

Figure S2. CP does not induce stress granules formation. Immunofluorescence analysis of the SG marker G3BP1 in HEK293 cells. Cells were treated with 0.5 mM sodium arsenite ($NaAsO_2$) for 15 min to induce SG formation. No redistribution of G3BP1 was observed in cells treated with 20 μM CP for 15 h. Nuclear DNA was stained with DAPI (left panel). Enlargements of boxed regions depicted in the merged images are shown in the right panel. Scale bar: 30 μm.

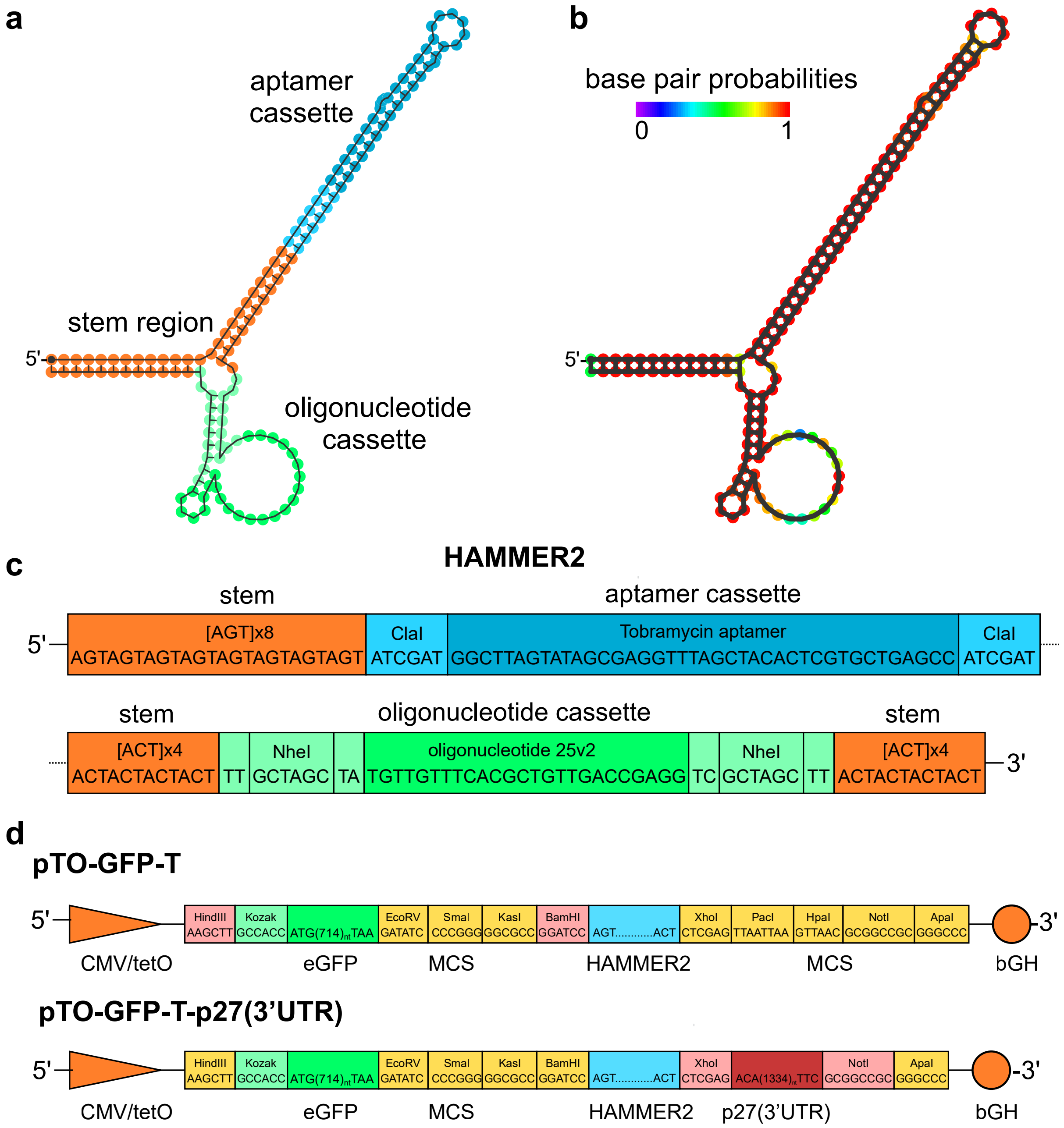

Figure S3. The HAMMER2 affinity tag and plasmids. (a) Centroid secondary structure of the HAMMER tandem affinity tag as predicted by RNAfold (81). The tobramycin aptamer (highlighted in blue) is flanked by sequences predicted to form a stable stem-structure (orange) to expose the RNA aptamer, and an oligonucleotide cassette oligonucleotide (green) that could be used for enrichment with ASOs (not used here). (b) Nucleotides are colour-coded according to base-pair probabilities (RNAfold). (c) Schematic representation of the HAMMER2 tandem affinity tag, showing the linker stem regions (orange), the tobramycin aptamer cassette (blue), and the oligonucleotide cassette (oligonucleotide: 25v2; green), together with the corresponding DNA sequence in the sense direction. Restriction sites that can be used to exchange the oligonucleotide cassettes (*Cla*I and *Nhe*I, respectively) are indicated. (d) Plasmids containing the HAMMER2 tandem affinity tag for generation of stable cell lines. The plasmids contain a chimeric tet operon/CMV minimal promoter (CMV/tetO; orange), allowing the tet-inducible expression of HAMMER2-containing transcripts in mammalian cells (arrows). Transcription is terminated by the bovine growth hormone (bGH) terminator. The eGFP expression cassette including the Kozak sequence (dark and light green, respectively), the p27(3'UTR) (dark red), and multiple cloning sites (MCS; yellow) to facilitated exchange of aptamer tags and 3'UTRs are indicated. The pTO reporter plasmids contains a Flippase recognition target (FRT) site (not shown), allowing recombinase-mediated single site genomic integration into a range of commercially available cell-lines.

81. Gruber, A.R., Lorenz, R., Bernhart, S.H., Neubock, R. and Hofacker, I.L. (2008) The Vienna RNA websuite. *Nucleic acids research*, **36**, W70-74.

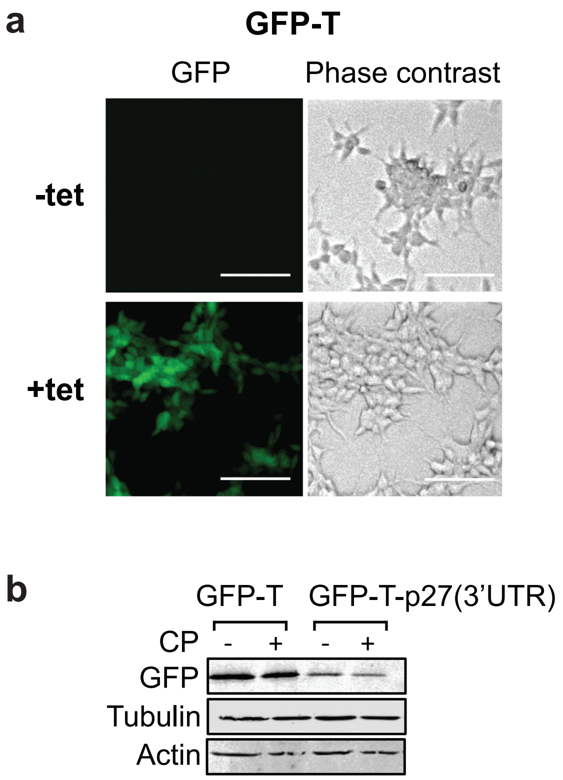

Figure S4. Inducible expression of GFP reporters in stable cell lines. (a) Phase contrast and fluorescence microscopy analysis of GFP-T and GFP-T-p27(3'UTR) cells treated with 1 µg/ml of tet for 24 h to induce GFP expression (+tet). Uninduced cells (-tet) are shown for comparison. Scale bar: 100 µm. (b) Immunoblot analysis of total lysate of the indicated cell lines, each treated for 48 h with 1 µg/ml tet and 20 µM CP added (+CP), or not added (-CP) for the last 15 h. The membrane was probed with antibodies directed against the indicated proteins.

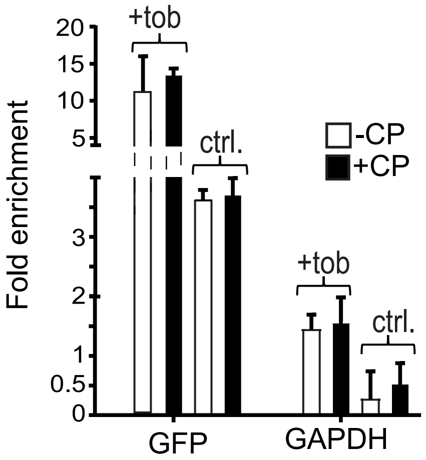

Figure S5. Enrichment of GFP-T-p27(3'UTR) in tobramycin affinity eluates. Relative enrichment of GFP-T-p27(3'UTR) and GAPDH (control) mRNAs was determined by RT-qPCR normalised to tubulin as compared to the input. RNA isolations were performed from CP treated (white bar) and untreated (black bar) cells. A control (ctrl.) was performed with beads devoid of tobramycin (n=3, SEM).

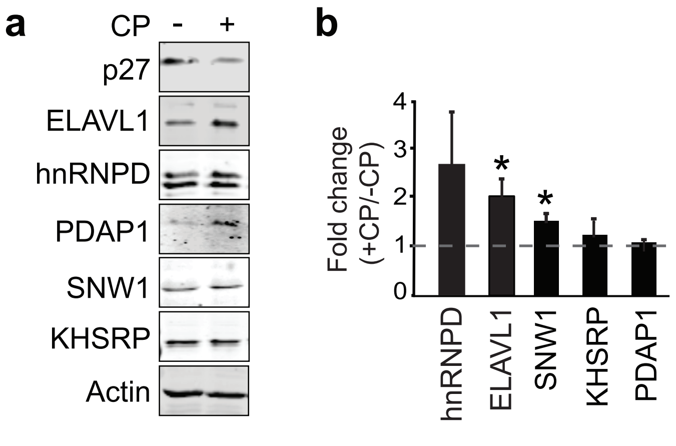

Figure S6. Expression of selected RBPs upon CP treatment of cells. HEK293 cells were treated for 15 h with 20 µM CP. (a) Immunoblot analysis antibodies directed against the indicated proteins on total cell lysates. (b) Fold changes of respective mRNAs in CP-treated compared to untreated cells determined by RT-qPCR. Data was normalised to *β-actin* (n=3, SEM).

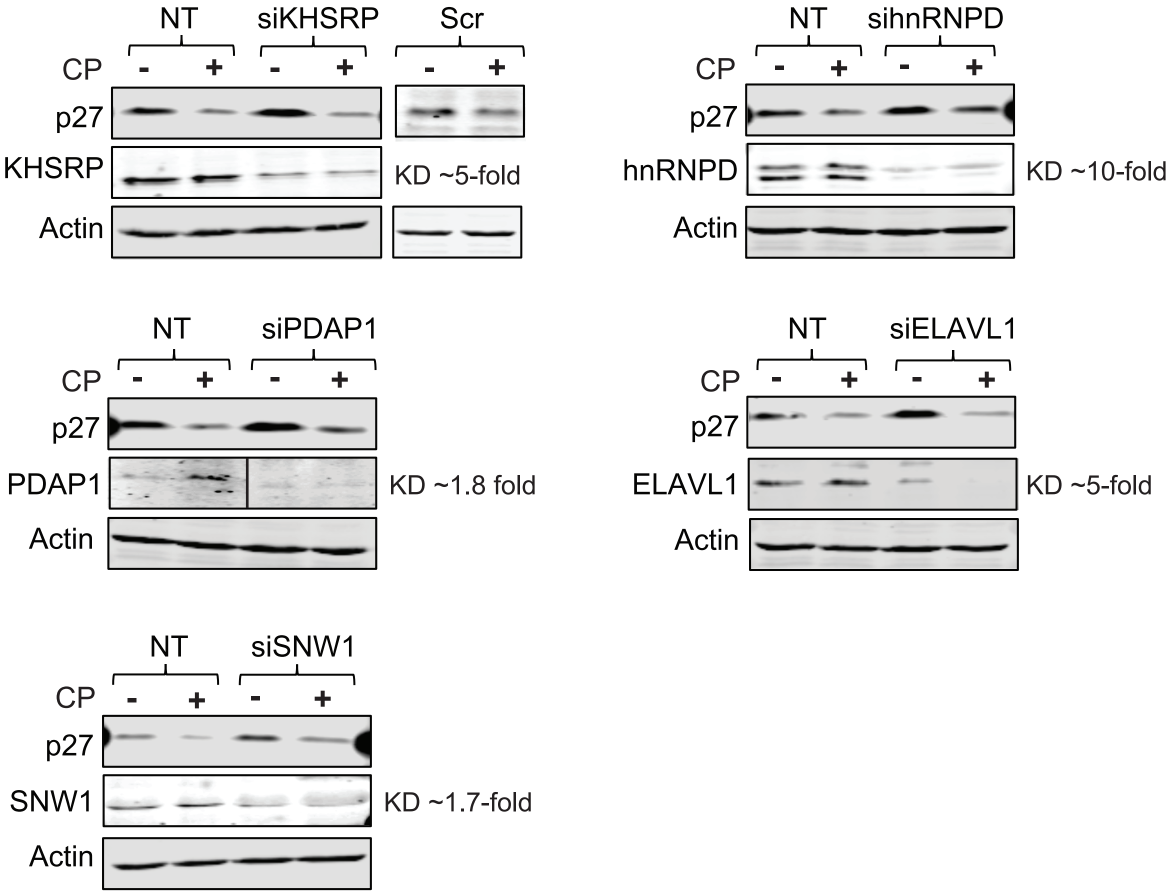

Figure S7. Changes of p27 protein levels upon knock-down of selected RBPs. Representative immunoblots detecting p27 protein and indicated RBPs in cell lysates prepared from non-transfected (NT) and siRNA transfected cells (indicated on the top), which were treated (+CP) or not (-CP) with 20 µM CP for the last 15 h. The levels of RPBs were not specifically changed in control siRNA (Scr) cells as compared to NT cells (data not shown). The average knock-down (KD) efficiency (fold-change) is indicated to the right of the RBP. Parts of the figure referring to the expression of RBPs in untreated cells is also displayed in Supplemental Figure S6.
