# Supplementary figures and images for: Tandem RNA isolation reveals functional rearrangement of RNA-binding proteins on CDKN1B/p27Kip1 3’UTRs in cisplatin treated cells
Source: RNA Biol. 2019 Sep 16;17(1):33–46. doi: 10.1080/15476286.2019.1662268 (PMC6948961; doi:10.1080/15476286.2019.1662268)

**Supplemental Materials**

**Uncropped immunoblots.** (a) Fig. 1d, (b) Fig. 3b, (c) Fig. 5a, (d) Fig. 5c.

**a**

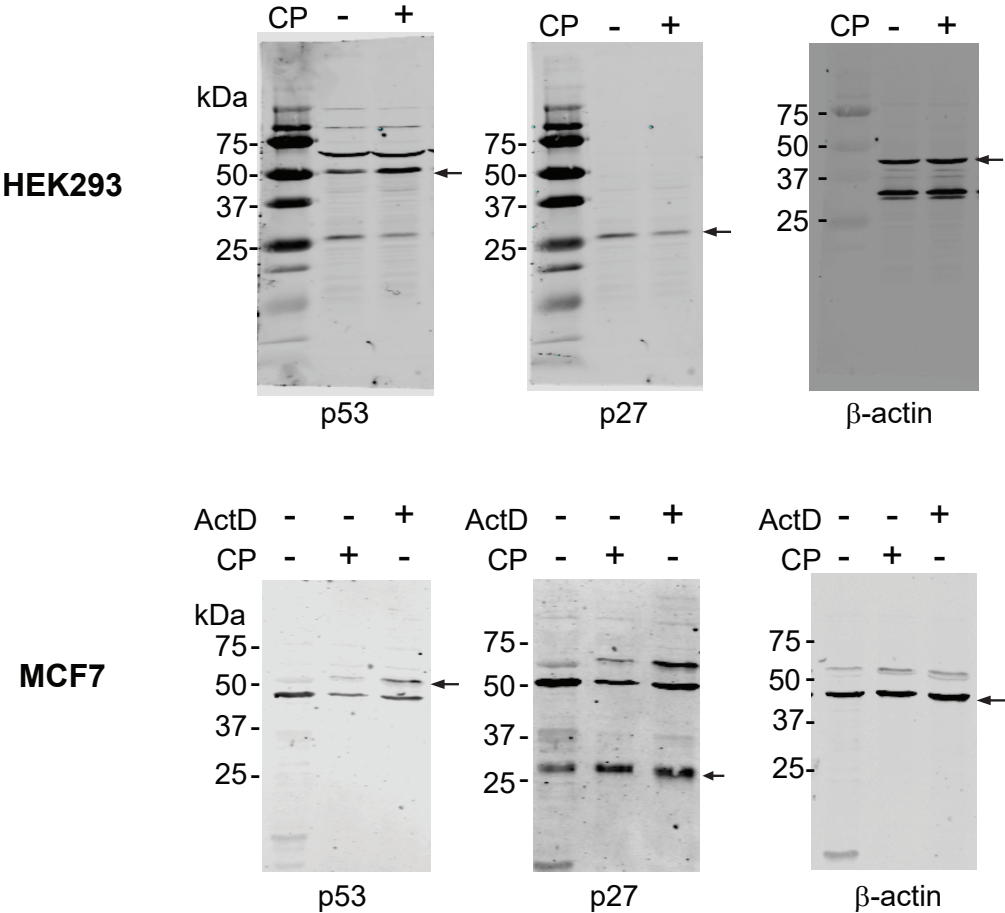

**b**

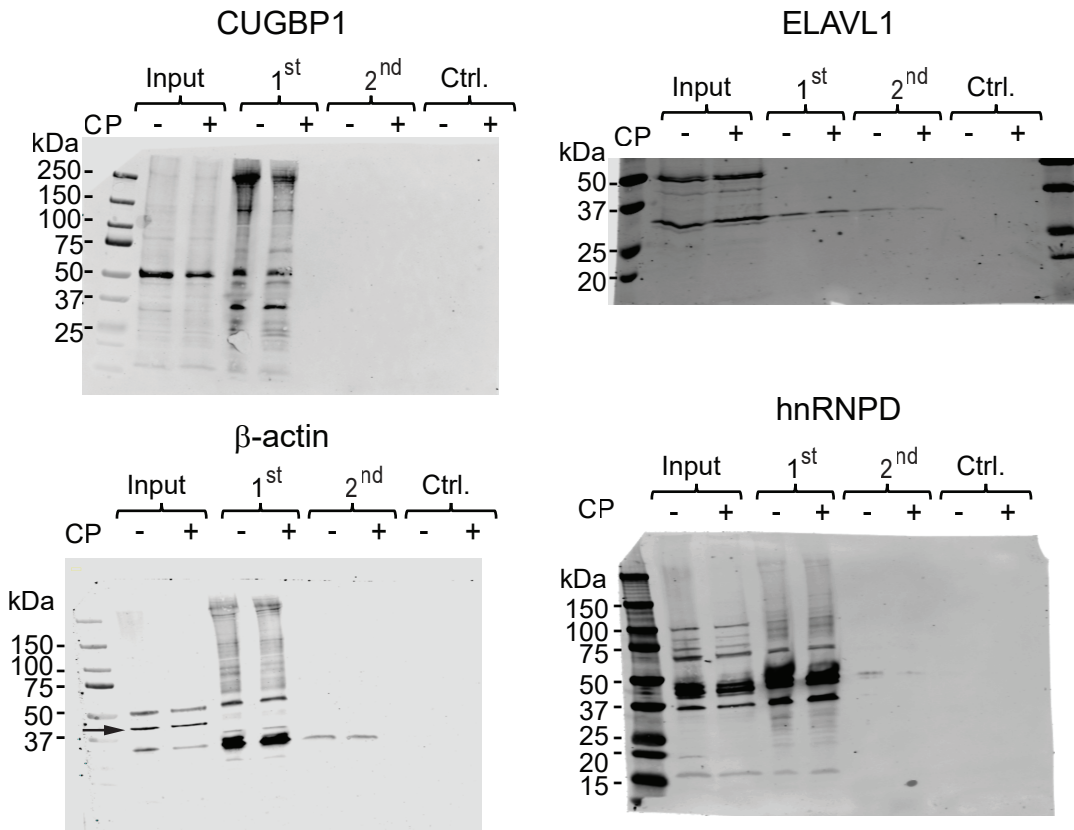

**c**

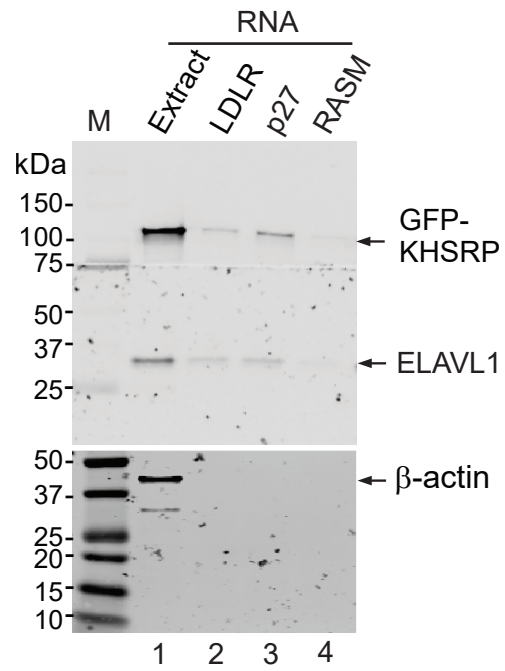

**d**

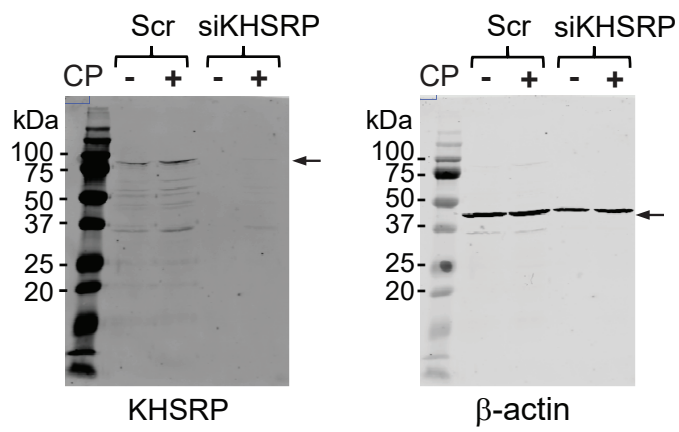

Supplement: Supplemental Material [file krnb-17-01-1662268-s001.zip › Supplementary information/Uncropped immunoblots_Iadevaia.pdf]
